# Poor glycemic control can lead to an early appearance of atherosclerosis in patients with type 1 diabetes - Can this be avoided by effective educational programs?

**DOI:** 10.1590/2359-3997000000304

**Published:** 2017-12-01

**Affiliations:** 1 Universidade Federal do Rio de Janeiro Faculdade de Medicina Rio de Janeiro RJ Brasil Faculdade de Medicina da Universidade Federal do Rio de Janeiro (UFRJ), Rio de Janeiro, RJ, Brasil

This issue of “*Archives of Endocrinology and Metabolism*” features two papers about type 1 diabetes mellitus (T1DM). Pacheco and cols. report the results of a structured education program for adults with T1DM from a single center in the south of Brazil. The education strategy consisted of workshops, individualized care and 24-hour distant support (1). The design of the program was inspired by the educational support provided in the Dose Adjustment for Normal Eating Study (DAFNE), performed in the UK (2). Fortunately, the authors report successful results, leading to a significant improvement in HbA1c (approximately 20% in one year with an additional reduction of 11% in the next 8 months) (1). Achieving the HbA1c goals in individuals with T1DM is still a major challenge worldwide. Although adults tend to have a slightly better glycemic control than younger patients, the proportion of individuals with 25 years of age or more who present HbA1c below 7.5% still varies between 20.5% and 53.6% in different countries, with a mean HbA1c between 8% and 8.5% in most populations that provide enough resources for diabetes care (3). A mean HbA1c of 9.2% has been previously reported for the Brazilian population, where the resources for diabetes care are limited and efficient national programs for diabetes care are still lacking (4).

Pacheco and cols. (1) reported a mean HbA1c of approximately 10.7% in males and 9.6% in females before the initiation of the educational program in one center in south Brazil. The authors achieved a mean HbA1c of approximately 8.5% for both men and women in the end of the study, which is still above the recommended goals for T1DM but at the same range obtained for countries that offer more resources for diabetes treatment and glucose monitoring than Brazil. A control group that did not participate in the educational program did not show any differences in the glycemic control in a similar time period, which reinforces the impact of the intervention in this study group. These results indicate that education can be an effective tool to obtain significant improvements in diabetes care in patients with T1DM, including poor countries with limited health-care resources.

Although the success of educational intervention programs had been reported in previous studies, including the DAFNE study itself (2,5), this is not a rule. A meta-analysis failed to demonstrate strong benefits of educational programs in the improvement of glycemic control in patients with T1DM from different populations (6). As educational programs can vary significantly, the lack of success in achieving better glycemic control with such intervention might indicate the choice of an inadequate program or an erroneous selection of the intervention group. Therefore, successful models of educational programs for diabetes care, such as the one reported by Pacheco and DAFNE, should be replicated and further studied, in order to be included as part of the routine health care policies for patients with diabetes. This can represent a simple and cheap intervention to improve glycemic control in patients with T1DM worldwide, especially in poor countries.

Improving glycemic control in people with T1DM is particularly important as it is known that this can reduce the risk of diabetic chronic complications (7). The Diabetes Control and Complications Trial (DCCT)/Epidemiology of Diabetes Interventions and Complications (EDIC) Trial has shown that intensive insulin treatment aiming to achieve HbA1c < 7% has beneficial effects in the risk reduction not only of micro vascular complications (8), but also of macrovascular complications (9). After the completion of the DCCT, which compared intensive therapy (IT) with a conventional therapy (CON) in the risk of chronic complications, patients were invited to take part of the EDIC Trial. This was an observational study where IT was offered to all participants that completed DCCT, independently of the previous group, due to the significant impact obtained with IT in the risk reduction of diabetic complications. Although the mean HbA1c significantly differed between the IT and CON groups during the DCCT, both groups had similar mean HbA1c levels during the EDIC trial. Nevertheless, the benefits of the intensive control observed during the DCCT not only persisted but also became more prominent during the EDIC, indicating the existence of a “metabolic memory” and showing the importance of maintaining a good glycemic control since the beginning of the disease (10).

Also in this issue of “*Archives of Endocrinology and Metabolism*”., Tacito and cols. have shown that patients with T1DM had impaired endothelial function, when compared to healthy controls (11). The authors also compared the carotid artery intima- media thickness (CIMT) between patients with T1DM and a control group using an automated system. Both endothelial dysfunction and CIMT are early markers of atherosclerosis, which are implicated in the pathogenesis of macrovascular complications. Even though both groups described in the study performed by Tacito and cols. had CIMT values within the normal range for age, patients with T1DM had higher CIMT than controls. Curiously, the study group was young, had a short disease duration (of approximately 4 years), normal body mass index (BMI), but had a high mean HbA1c (of 9.95%) (11). It is possible that the poor glycemic control contributed to the precocious appearance of early atherosclerosis markers, even in this group of young lean patients with a short disease duration. This is consistent with the concept of “metabolic memory” and stresses the importance of starting an intensive treatment and achieving a good glycemic control since the beginning of the disease. Although endothelial function is closely related to T1DM duration, the early development of endothelial dysfunction in patients with T1DM, short disease duration and poor glycemic control had already been previously reported (12,13). On the other hand, alterations in CIMT have been reported mostly in patients with longer disease duration (14-16). In the EDIC, after 1 year of follow-up, the carotid intima- media thickness in patients with T1DM with a mean disease duration of approximately 14 years was similar to that in an age- and sex-matched nondiabetic population, both in CON and IT groups (17).

Another possible explanation for the findings seen in the study performed by Tacito and cols. (11) is the difference in TSH levels between the groups (patients vs controls). Several studies have reported a link between CIMT and subclinical hypothyroidism (18-20), but Takamura and cols. have also reported an association between TSH and CIMT in euthyroid individuals (21). Although TSH levels were within the normal range in both groups, the levels significantly differed between them. Indeed, it has been suggested that a narrower thyrotropin reference range should be used in the general population (22) as the current reference ranges have been defined in populations previously considered healthy but included individuals with various degrees of thyroid dysfunction. Moreover, we have previously found that TSH levels > 2.5 mU/l, even in the normal range, are associated with a higher risk of retinopathy and renal dysfunction in patients with T1DM than TSH levels between 0.4 and 2.5 mU/l (23). Possibly, the same association could be observed for macrovascular complications. This would be an alternative explanation for the finding obtained by Tacito and cols. (11) and worth further investigation, since thyroid diseases are more frequent in patients with T1DM than in the general population.

To conclude, the two interesting papers featured in this issue of “*Archives of Endocrinology and Metabolism*” bring us very important messages. Firstly, patients with T1DM and poor glycemic control might develop early signs of atherosclerosis even in the first few years of the disease, at a young age and with normal BMI. Secondly, efficient educational programs can lead to a significant reduction of HbA1c levels in patients with T1DM, which can lead to a significant risk reduction of micro and macrovascular diabetic complications. Therefore, effective educational programs should be implemented soon after the diagnosis of T1DM, in order to avoid poor glycemic, metabolic memory and their possible adverse effects.
